# Density‐weighted concentric circle trajectories for high resolution brain magnetic resonance spectroscopic imaging at 7T

**DOI:** 10.1002/mrm.26987

**Published:** 2017-11-06

**Authors:** Lukas Hingerl, Wolfgang Bogner, Philipp Moser, Michal Považan, Gilbert Hangel, Eva Heckova, Stephan Gruber, Siegfried Trattnig, Bernhard Strasser

**Affiliations:** ^1^ High Field MR Centre, Department of Biomedical Imaging and Image‐guided Therapy Medical University of Vienna Vienna Austria; ^2^ Christian Doppler Laboratory for Clinical Molecular MR Imaging Medical University of Vienna Vienna Austria

**Keywords:** magnetic resonance spectroscopic imaging, 7T, spatial‐spectral encoding, non‐cartesian trajectory, concentric circles, density‐weighted acquisition

## Abstract

**Purpose:**

Full‐slice magnetic resonance spectroscopic imaging at ≥7 T is especially vulnerable to lipid contaminations arising from regions close to the skull. This contamination can be mitigated by improving the point spread function via higher spatial resolution sampling and k‐space filtering, but this prolongs scan times and reduces the signal‐to‐noise ratio (SNR) efficiency. Currently applied parallel imaging methods accelerate magnetic resonance spectroscopic imaging scans at 7T, but increase lipid artifacts and lower SNR‐efficiency further. In this study, we propose an SNR‐efficient spatial‐spectral sampling scheme using concentric circle echo planar trajectories (CONCEPT), which was adapted to intrinsically acquire a Hamming‐weighted k‐space, thus termed density‐weighted‐CONCEPT. This minimizes voxel bleeding, while preserving an optimal SNR.

**Theory and Methods:**

Trajectories were theoretically derived and verified in phantoms as well as in the human brain via measurements of five volunteers (single‐slice, field‐of‐view 220 × 220 mm^2^, matrix 64 × 64, scan time 6 min) with free induction decay magnetic resonance spectroscopic imaging. Density‐weighted‐CONCEPT was compared to (a) the originally proposed CONCEPT with equidistant circles (here termed e‐CONCEPT), (b) elliptical phase‐encoding, and (c) 5‐fold Controlled Aliasing In Parallel Imaging Results IN Higher Acceleration accelerated elliptical phase‐encoding.

**Results:**

By intrinsically sampling a Hamming‐weighted k‐space, density‐weighted‐CONCEPT removed Gibbs‐ringing artifacts and had in vivo +9.5%, +24.4%, and +39.7% higher SNR than e‐CONCEPT, elliptical phase‐encoding, and the Controlled Aliasing In Parallel Imaging Results IN Higher Acceleration accelerated elliptical phase‐encoding (all P < 0.05), respectively, which lead to improved metabolic maps.

**Conclusion:**

Density‐weighted‐CONCEPT provides clinically attractive full‐slice high‐resolution magnetic resonance spectroscopic imaging with optimal SNR at 7T. Magn Reson Med 79:2874–2885, 2018. © 2017 The Authors Magnetic Resonance in Medicine published by Wiley Periodicals, Inc. on behalf of International Society for Magnetic Resonance in Medicine. This is an open access article under the terms of the Creative Commons Attribution License, which permits use, distribution and reproduction in any medium, provided the original work is properly cited.

## INTRODUCTION

Proton magnetic resonance spectroscopic imaging (MRSI) is a powerful non‐invasive imaging tool that is nowadays applied in neuroscience and clinical assessment of several major brain disorders alike 1. Due to improvements in both sensitivity and spectral separation of metabolite resonances, MRSI is one of the MRI methods that should particularly benefit from ultra‐high static magnetic field strength ( $B_{0}\geq 7$ T), but technical challenges associated with *B*_0_/*B*_1_ inhomogeneites, chemical shift displacement errors, specific absorption rate limits, and water/lipid suppression have long prevented a widespread application in patient studies 2, 3, 4, 5, 6. Only recent preliminary clinical applications of MRSI have been shown at 7T 7. This can be attributed to the advent of promising new MRSI approaches such as free induction decay (FID)‐MRSI 3, 4, 5. For FID‐MRSI the combination of high spatial resolution and spatial low‐pass filtering has led to substantial improvements of the point spread function (PSF), and hence a reduction of lipid artifacts, which enabled high‐resolution MRSI with full‐slice coverage, but at the expense of prolonged scan times 1. This has triggered the need for MRSI acceleration methods suitable for $B_{0}\geq 7$ T. At $B_{0}\leq 3$ T, MRSI acceleration is dominated by spatial‐spectral encoding (SSE) via echo‐planar spectroscopic imaging (EPSI) and spiral spectroscopic imaging, which offer acceleration factors of up to two orders of magnitude 8, 9. Yet, only a single article on SSE has been published so far at 7T using EPSI with a non‐standard head‐only gradient insert 10. Rather, MRSI acceleration at 7T and 9.4T has been dominated by simple reduction in repetition time (TR) 11, 12 and parallel imaging (e.g., sensitivity encoding 13, 14, generalized autocalibrating partially parallel acquisition 2, and Controlled Aliasing In Parallel Imaging Results IN Higher Acceleration [CAIPIRINHA] 15). This is a result of the gradient hardware limitations of SSE methods. Both, increased spatial resolution and spectral bandwidth, lead to increased demands on gradient systems and ultimately lower signal‐to‐noise ratio (SNR) efficiency 16, 17. Parallel imaging, on the other hand, becomes more efficient at higher *B*_0_, but achievable accelerations are lower than for SSE ( ≤10) 15 and lipid contamination is aggravated, which calls for additional lipid suppression techniques 2, 18, 19. While established SSE trajectories (i.e., EPSI, spirals) can be certainly optimized for higher *B*_0_, the increased gradient demands at 7T due to the increased spectral bandwidth make these trajectories inefficient for high spatial resolutions. This has triggered the development of alternative self‐rewinding SSE approaches including rosettes 20, 21 and concentric circle echo planar trajectories (CONCEPT; in the following termed e‐CONCEPT, because of equidistant circles) 22, 23, 24. Since these trajectories are self‐rewinding, no time is wasted for rewinding the trajectory after each spectral time point, making them very efficient. However, in contrast to EPSI and constant‐density spirals, which sample k‐space fairly uniformly, e‐CONCEPT features a k‐space density resembling that of a spatial low pass filter. As shown by Kasper et al., the SNR efficiency of a k‐space trajectory is highest if its density matches the target filter 25, 26. Therefore, e‐CONCEPT is favorable if the target k‐space density is a spatial low‐pass filter, but is inefficient for other target filters, such as a uniform k‐space density. Low‐pass filters such as Hamming or Hanning filters are particularly desirable for improving the PSF. In general, by spending more sampling time in specific k‐space regions, density‐weighting (DW) of k‐space can be achieved. Ideally, the target filter can be not only approximated by the natural density of the trajectory, but exactly matched. So far, Hanning weighting has been achieved via gradient slew‐rate demanding variable‐density spirals at 3T 27, which are unsuitable for $B_{0}\geq 7$ T. EPSI can be hardly adapted to low‐pass filtering. Rosettes have also unfavorable contributions of high‐pass filters 20, 21, although this is subject to optimization. The natural density of e‐CONCEPT is not a perfect Hamming filter per se, but is close. Also, e‐CONCEPT can be efficiently tuned to a Hamming or any other radially symmetric low‐pass filter function by distributing non‐equidistant circles accordingly, but neither with the increased gradient slew rate demands of variable‐density spirals 27 nor the excessively long scan times of acquisition weighting via elliptical phase encoding (ePE) 17, 28. This entirely avoids the inevitable SNR efficiency loss associated with retrospective k‐space filtering. The purpose of our study was, therefore, to develop DW‐CONCEPT trajectories with ideal Hamming k‐space weighting for high‐resolution (i.e., 64 × 64 matrix) 7T FID‐MRSI and to compare this approach with full ePE (gold standard) as well as 5‐fold CAIPIRINHA‐accelerated ePE with the same scan time of less than 6 min.

## THEORY

### SNR Efficiency

As shown by Kasper et al. 25, the noise variance $|\sigma_{\text{Acq}}{|}^{2}$ is given by

(1) $$|\sigma_{\text{Acq}}{|}^{2}=\int_{V_{k}}d^{n}k\frac{\rho_{\text{Target}}^{2}}{\rho_{\text{Acq}}}$$

when the k‐space is acquired with the density *ρ*_Acq_ and the target k‐space density is *ρ*_Target_. The SNR of such an acquisition relative to a uniform k‐space density is then given by:

(2) $$\frac{{\text{SNR}}_{\text{Acq}}}{{\text{SNR}}_{\text{Uniform}}}=\sqrt{\frac{|\sigma_{\text{Uniform}}{|}^{2}}{|\sigma_{\text{Acq}}{|}^{2}}},$$

where $|\sigma_{\text{Uniform}}{|}^{2}$ is the noise variance of a uniform k‐space density which can be calculated by setting $\rho_{\text{Acq}}=\rho_{\text{Uniform}}$ in Eq. (1). It is easy to show that SNR_Acq_, and thus Eq. (2) is maximized if $\rho_{\text{Acq}}=\rho_{\text{Target}}$ 25. For the case of a Hamming target density ( $\rho_{\text{Target}}=H$) and DW‐CONCEPT to exactly achieve this filter ( $\rho_{\text{Acq}}=H$), Eq. (2) evaluates to 125.2%, see Appendix A. For e‐CONCEPT the density is given by $\rho_{e-\text{CONCEPT}}=d_{1}/k$, where *d*_1_ is a normalization constant, and *k* is the k‐space radius 29. Using that, Eq. (2) evaluates to 114.9% if $\rho_{\text{Target}}=H$ and $\rho_{\text{Acq}}=\rho_{e-\text{CONCEPT}}$, which agrees with previous studies 23. Therefore, e‐ and DW‐CONCEPT are more efficient than sequences with a constant k‐space density, such as ePE, if the target density is Hamming. Furthermore it follows that the SNR of DW‐CONCEPT is 109.0% the SNR of e‐CONCEPT.

### DW‐CONCEPT

Since SNR efficiency is highest when $\rho_{\text{Acq}}=\rho_{\text{Target}}$, the question arises how to distribute the radii of e‐CONCEPT circles to achieve a Hamming density. For this, we need to define the k‐space density which is the ratio of the sampling duration ΔTime to the occupied k‐space area:

(3) $$\rho_{\text{Acq}}\propto \frac{\Delta \text{Time}}{\Delta k‐\text{Space area}}.$$

If the analog to digital converter dwell time and the angular velocity of each circle is kept constant, ΔTime is a constant. Assuming further a continuous k‐space, the k‐space area assigned to a k‐space point can be described by the Jacobian determinant *J* 29, 30, 31 of the coordinate transformation

(4) $$\left(k_{x},k_{y})=(K(k)\cos k_{\phi },K(k)\sin k_{\phi }\right)$$

resulting in $J=K\frac{dK}{dk}$. *K*(*k*) describes an arbitrary radii distribution of the circles and translates from an equidistant set of k‐space radii *k*, to a non‐uniform radii distribution *K*(*k*). By calculating this function, the desired k‐space density can be achieved. It is required to fulfill

(5) $$\begin{array}{l} K(0)=0\\ K(k_{\text{max}})=k_{\text{max}}, \end{array}$$

where $k_{\text{max}}$ is the radius of the largest circle to achieve a given resolution and FOV. As an example, the density of equidistant circles is obtained by setting *K*(*k*) = *k*: $\rho_{e‐\text{CONCEPT}}=1/J\propto 1/k$.

**Figure 1:**
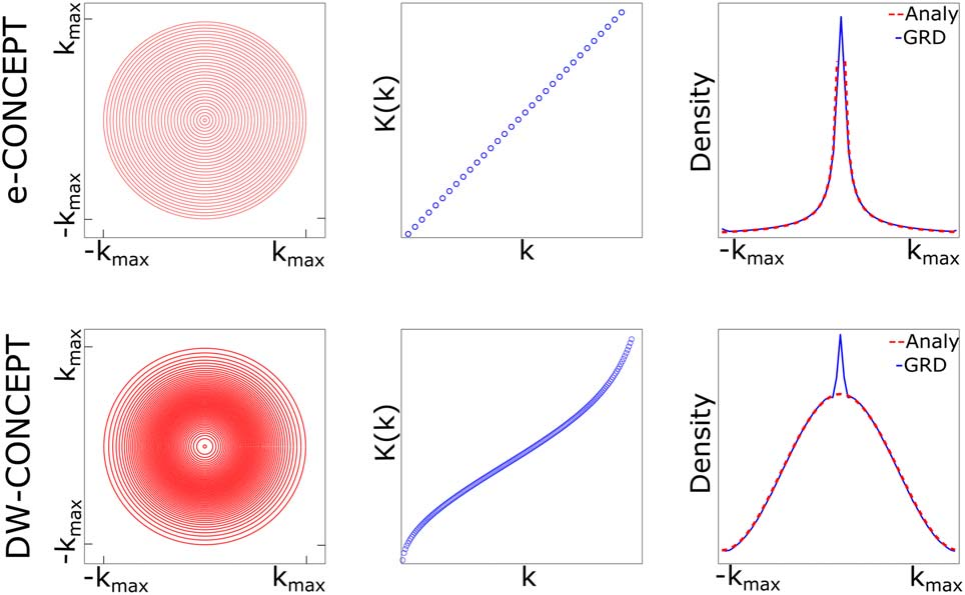
From left to right: The distributions of the circles (first column), given by *K*(*k*) (second column) and the associated densities for e‐CONCEPT (equidistant radial increments) and DW‐CONCEPT (variable radial increments), third column. The radii $K(k_{i})$ can be derived by discretization of the horizontal axis. This results in a 1/k‐density for e‐CONCEPT and a Hamming density for DW‐CONCEPT. The density simulations (blue, 3rd column) have been performed by gridding of ones without density compensation and an overgridding factor of 1. They match the analytical solutions (red), except of the peaks in the k‐space center which result from imperfections of the gridding algorithm. However they can be taken care of via the pre and post gridding density compensation. We observed a maximum number of three points deviating stronger from the expected densities in the center. For DW‐CONCEPT very minor deviations of the density along the outer most circle (radius *k*_max_) were observed due to the Taylor approximation of *K*.

In contrast, if we demand $\rho_{\text{Acq}}=\rho_{\text{DW}‐\text{CONCEPT}}$ it follows $H(K(k))=\alpha +\beta \cos(\pi K(k)/k_{\text{max}})=c_{1}/J$ and we obtain

(6) $$\frac{dK(k)}{dk}K(k)=\frac{c_{1}}{\alpha +\beta \cos(\pi K(k)/k_{\text{max}})},$$

with α=0.54 and β=0.46. This differential equation can be solved by separation of variables (see Appendix B), yielding the function *k*(*K*). Yet, the inverse function, *K*(*k*), describes the distribution of the circle radii to achieve a Hamming filter, and is thus required. Although the resulting function k=k(K) cannot be solved analytically, an inverse series expansion can be performed up to an arbitrary order. For this work, the function *K*(*k*) was approximated up to the 70th order. Evaluating *K* for discrete *k_i_*, $i=1,\ldots ,N_{\text{min}}$, *N*_min_ number of circles determined by the Nyquist criterion, gives the radii distribution $K(k_{i})$ to achieve a Hamming density, see Figure 1. The number of circles needed to achieve the 1/k and the Hamming weighting are displayed in Table 1 for various matrix sizes.

**Table 1:** The Minimum Number of Circles *N*_min_ Needed In Order to Sample a 1/k (e‐CONCEPT) or a Hamming‐Weighted (DW‐CONCEPT) k‐Space for the Matrix Sizes Above, were *N*_min_ is Determined by the Radial Nyquist Criterion

	e‐CONCEPT	DW‐CONCEPT
*N*_min_ for 32 × 32	16	44
*N*_min_ for 64 × 64	32	177
*N*_min_ for 128 × 128	64	714
TA for 64 × 64	40s	5 min 25 s

## METHODS

### Subjects and Hardware

A silicone oil phantom and five healthy volunteers were measured on a 7T MR system (Magnetom, Siemens Healthcare, Erlangen, Germany) with a 32‐channel head coil (Nova Medical, Wilmington, MA). A volume coil in the same housing was used for signal excitation. The gradient coils were capable of a maximum nominal amplitude of 40 mT/m and a maximum slew rate of 200 mT/m/ms. The local institutional review board approved this study, and written consent was obtained from all volunteers.

### Sequence Description

**Figure 2:**
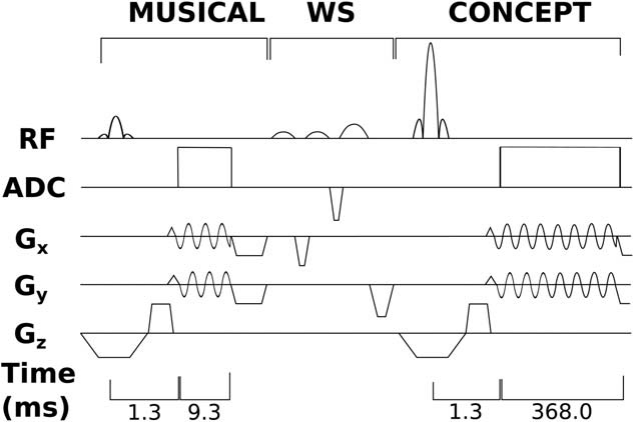
Simplified pulse sequence diagram consisting of the MUSICAL prescan (acq. dur. 9.3 ms), Water‐suppression enhanced through *T*_1_‐effects water suppression and the 2D FID‐MRSI sequence with CONCEPT readout (acq. dur. 368.0 ms, acq. delay 1.3 ms, TR = 600 ms). Due to three temporal interleaves, each three excitations in a row describe the acquisition of a certain k‐space radius until the next one is acquired. Not denoted is the initial noise decorrelation scan and four preparation scans.

All used MRSI sequences are based on a single‐slice FID sequence 3, 4 with a three‐lobe sinc excitation pulse. Water‐suppression enhanced through *T*_1_‐effects was used prior to signal acquisition. A short pre‐scan for acquiring noise‐only data was implemented into the ePE‐ and both CONCEPT sequences. MUSICAL coil combination 32 weights were measured using a gradient echo pre‐scan in case of ePE, or by playing out the same trajectory as in the actual scan for both CONCEPT sequences. This additional measurement was done before water suppression and with a low flip angle of 5°. For both, e‐ and DW‐CONCEPT, a gradient pre‐winder was necessary to (a) reach the intended k‐space radius, and (b) acquire the transverse velocity for enabling one circumnavigation within the desired spectral dwell time. Although faster pre‐winders are possible, a simple solution was chosen: The trajectory first moves radially outward, and accelerates then tangentially along the circle. Afterward, several circumnavigations of the same circle are performed with a constant angular velocity, each corresponding to one FID point. After acquiring all FID points, the gradients have to be ramped down in order not to violate slew rate restrictions. Finally, a different circle is measured in another repetition time. A sequence diagram is shown in Figure 2. In total, four MRSI sequences with identical slice selection, matrix size, sequence timing, water suppression and PSF (see “Reconstruction” section), but different spatial‐spectral encoding were compared:

1. e‐CONCEPT: As originally proposed by Furuyama et al. 22, the circle radii for e‐CONCEPT were increased with a constant step size of 1/FOV, starting with the smallest circle of radius $K(k_{1})=k_{1}=1/(2$FOV).
2. DW‐CONCEPT: In contrast, the number of circles *N* for DW‐CONCEPT can be set by the user based on scan time restrictions, where $N\geq N_{\text{min}}$ according to the radial Nyquist criterion. The radii distribution for DW‐CONCEPT is given in Appendix B. Also here the first circle has the radius $K(k_{1})=1/(2$FOV). The sampling in azimuthal direction for both CONCEPT encodings was performed as described in Ref. 22, 33.
3. ePE: To compare both CONCEPT approaches with a gold standard, conventionally phase encoded MRSI of a circular k‐space was acquired.
4. CAIPIRINHA‐ePE: Retrospective undersampling of the fully acquired ePE scan with a 2D‐CAIPIRINHA pattern 15 with an acceleration factor of 5 was performed to match the scan time of both CONCEPT sequences.

### In Vivo and Phantom Measurements

A magnetization‐prepared 2 rapid acquisition gradient echoes 34 sequence was measured for anatomical reference with a nominal resolution of 0.9 × 0.9 × 1.1 mm^3^ within 5 min. To achieve the intended flip angle of 45° for the MRSI measurements, a $B_{1}^{+}$‐map was acquired with a pre‐saturation turboFLASH‐based *B*_1_ mapping sequence 35, 36. The *B*_0_ shim volume was placed to cover a ≈4 cm thick slab, including the subcutaneous lipid layer. All four compared MRSI sequences had the following consistent scan parameters: TR 600 ms, acquisition delay 1.3 ms, matrix size 64 × 64, FOV 220 × 220 mm^2^, and slice thickness 10 mm. The spectral bandwidth of ePE was 3000 Hz with 2‐fold oversampling and the scan time was 30 min 7 s. Retrospective undersampling via a 5‐fold accelerated 2D‐CAIPIRINHA pattern resulted in a simulated measurement time of 6 min 10 s, which was similar to that of both CONCEPT approaches with 5 min 52 s. For e‐CONCEPT, six averages were performed ( 6×32 circles), while DW‐CONCEPT was measured with one average (177 circles necessary for Nyquist sampling + 15 circles to match the scan time). 819 FID points were measured within three temporal interleaves resulting in a spectral bandwidth of 2778 Hz. A bandwidth of around 1800 Hz would be sufficient to cover the spectral range between 0 and 6 ppm. However, this caused baseline distortions and a lipid side lobe aliasing from −3 to 3 ppm. Therefore, a higher spectral bandwidth was chosen. To allow a reasonable comparison, the different spectral bandwiths were considered in the SNR calculations. The read‐out bandwidth was 250 kHz with total analog to digital converter lengths of 368 ms for CONCEPT and 341 ms for ePE. A bandwidth in the range of 950–1250 Hz for a single temporal interleave was deliberately avoided, because this range covers the forbidden acoustic resonances that could potentially harm our gradient system. The silicone oil phantom measurement was performed with the same protocol as the in vivo measurement, except that no magnetization‐prepared 2 rapid acquisition gradient echoes sequence was performed, and the water suppression was turned off.

### Reconstruction

For both, e‐ and DW‐CONCEPT, a modified Pipe‐Menon 37 pre‐gridding density compensation which density compensates to a Hamming weighted k‐space by choosing the appropriate weights, was applied. A post‐gridding density correction was used to remove small deviations from the targeted Hamming density due to gridding imperfections. Therefore no filtering was necessary in post‐processing for e‐ and DW‐CONCEPT. For DW‐CONCEPT, this process did not effectively change the density a lot, while for e‐CONCEPT the density was transformed from a 1/k‐density to a Hamming density. The chosen reconstruction approach resulted in a higher SNR compared to the usual way where the k‐space density is made constant before gridding and subsequent Hamming filtering (+4.9% for e‐CONCEPT and +6.8% for DW‐CONCEPT). A linear phase correction was applied prior gridding to compensate for the different acquisition times of each sample along the circles 38. This is necessary to avoid spatial blurring for off‐resonant signals. Gridding 39 using a Kaiser‐Bessel kernel (kernel width of 3) was performed with an overgridding factor of 2. After gridding, the data were spatially Fourier transformed and cropped to the intended FOV. For the CAIPIRINHA‐ePE data, the parallel imaging reconstruction was done in k‐space with a generalized autocalibrating partially parallel acquisition‐based algorithm as described by Strasser et al. 15. All four compared methods were further pre‐processed the same way apart from a final Hamming filtering for ePE and CAIPIRINHA‐ePE. Thus, all sequences had the same Hamming weighted k‐space at the end to ensure the same PSF. L2 lipid regularization 19 was additionally applied for volunteer #3 to show further potential improvements of the spectral baseline and metabolic map quality, but otherwise all data are presented without lipid removal to illustrate the excellent metabolic map quality obtained already without additional L2 lipid regularization.

### Evaluation

All spectra were processed using LCModel with basis sets of 17 brain metabolites as simulated via NMRScope (implemented into JMRUI 5.2) 40 and containing measured macromolecular backgrounds 41. SNR was computed using the pseudo‐replica method in the spectral domain 42. Linewidth was defined as the full‐width‐at‐half‐maximum of the fitted NAA resonance. Maps of metabolite levels, spectral quality (e.g., SNR, linewidth) and fitting precision (i.e., Cramer‐Rao Lower Bounds [CRLBs]) were created. Two‐tailed paired t‐tests with Bonferroni correction were performed on the mean SNRs of all voxels of each volunteer for all methods using IBM SPSS Statistics 22 (Armonk, NY). The means of the relative SNRs over all volunteers were calculated.

## RESULTS

### Phantom Results

**Figure 3:**
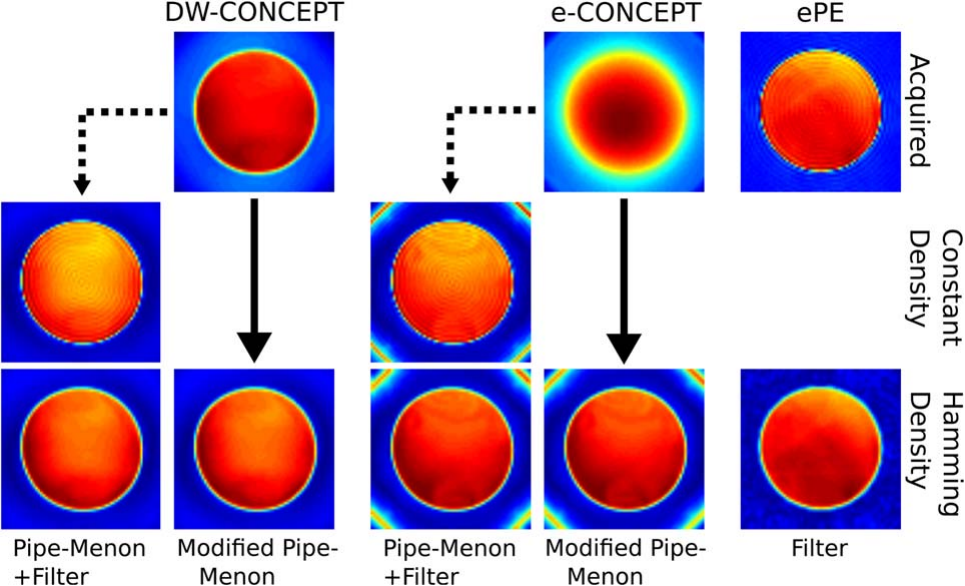
Oil phantom experiments measured with the in vivo protocol (FOV of 220 × 220 mm^2^, slice thickness 10 mm, 64 × 64 matrix) are shown. The used reconstruction for the CONCEPT sequences consisted of a modified Pipe‐Menon density compensation which directly allowed gridding on a Hamming weighted k‐space (solid arrow)—contrary to the classical approach (dashed arrow) which flattens k‐space prior gridding and applies retrospective filtering. The modified Pipe‐Menon approach resulted in additional SNR gains of ≈4.9% for e‐CONCEPT and ≈6.8% for DW‐CONCEPT when compared to the conventional Pipe‐Menon density compensation. For ePE and CAIPIRINHA‐ePE only filtering was performed. Hence, in the end all compared methods reached the same PSF.

The results of the silicone oil phantom measurement with the in vivo protocol are shown in Figure 3, displaying the localization performance of the three measured sequences. Apparently, e‐CONCEPT without density compensation results in bad localization, while DW‐CONCEPT does not. Also the polar sampling artifact due to the *Jinc*‐shaped PSF 29 is clearly visible, contrary to DW‐CONCEPT because of oversampling. ePE clearly shows significant Gibbs ringing artifacts, which can be reduced by retrospective filtering. Prior spatial Fourier transformation our modified Pipe‐Menon density compensation (for e‐ and DW‐CONCEPT only) and Hamming filtering (for ePE and CAIPIRINHA‐ePE only) were applied.

### In Vivo Results

**Figure 4:**
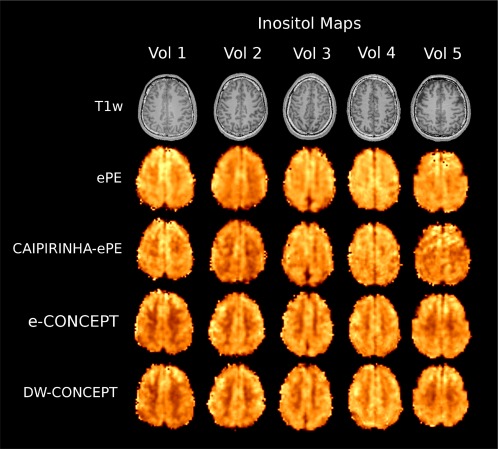
myo‐Inositol maps of five volunteers together with *T*_1_‐weighted images with the four compared methods ePE (30 min 7 s), CAIPIRINHA‐ePE (6 min 10 s), e‐ and DW‐CONCEPT (5 min 52 s). For all methods the same protocol was used: FOV of 220 × 220 mm^2^, slice thickness 10 mm, 64 × 64 matrix. No lipid regularization was used.

**Figure 5:**
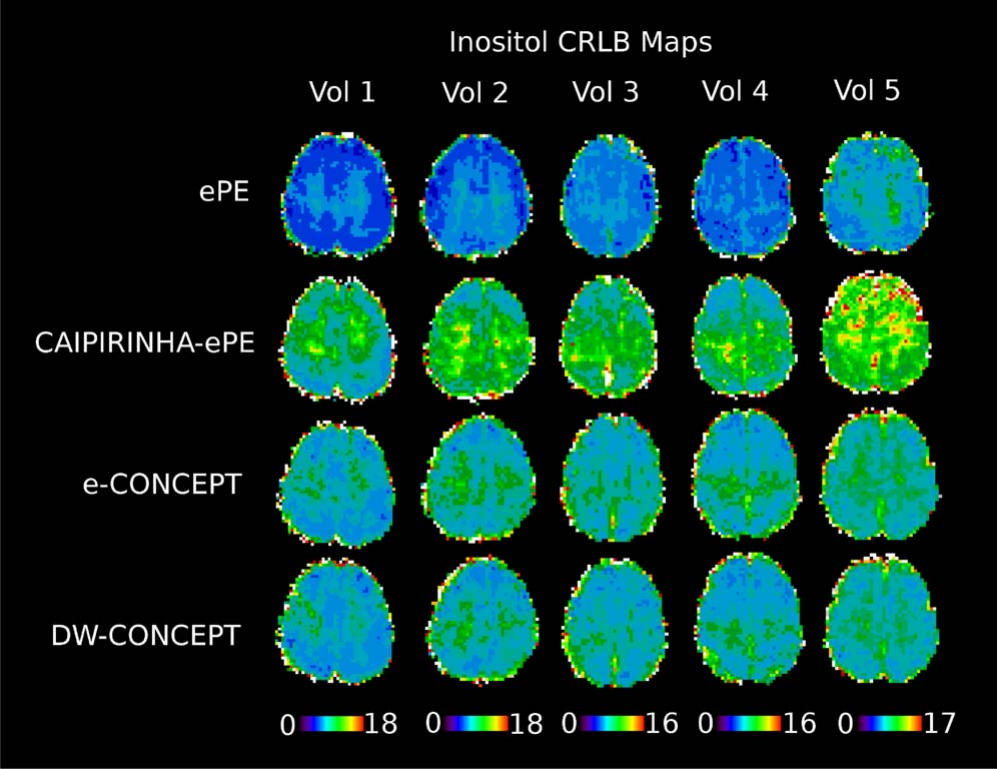
CRLB maps for inositol across all five volunteers and methods. CRLB values are taken from LCModel and as can be seen, DW‐CONCEPT performs best with the lowest errors (except for ePE, which had a five times longer measurement time).

**Figure 6:**
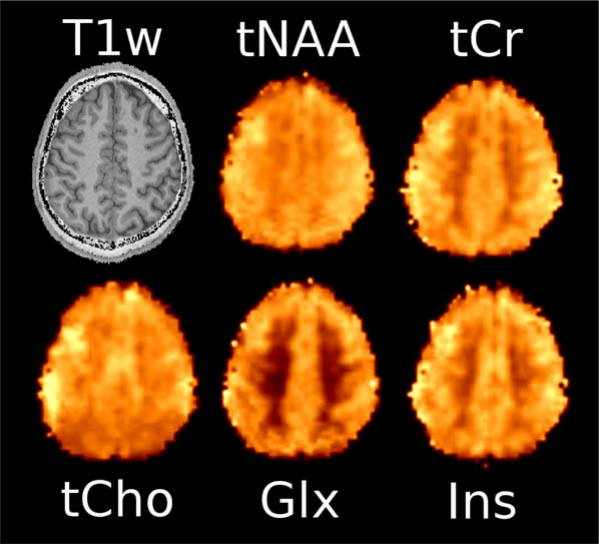
Metabolic maps of the second volunteer obtained with DW‐CONCEPT. Results for total N‐acetyl‐aspartate, total creatine, total choline, glutamine + glutamate, and inositol are shown.

**Figure 7:**
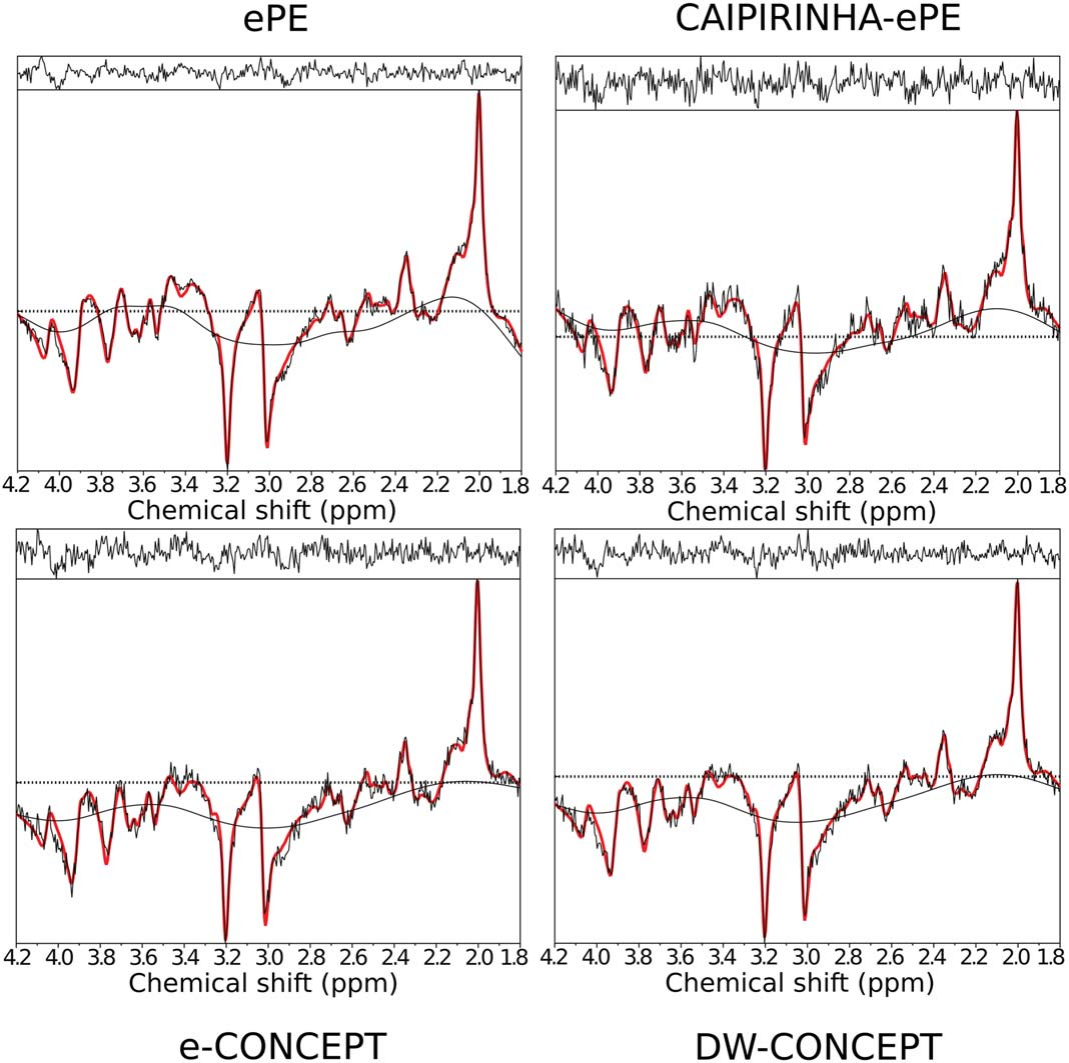
Comparison of spectra taken from a central gray matter voxel of the first volunteer without lipid regularization. Spectra were taken from LCModel and show the fitting (red) and the measured data (black). The CAIPIRINHA‐ePE spectrum exhibits more noise and a worse fitting than the other methods. For the CONCEPT sequences we observed baseline artifacts for voxels closer to the skull, which possibly result from unwanted lipid signals, see Supporting Figure S1.

**Figure 8:**
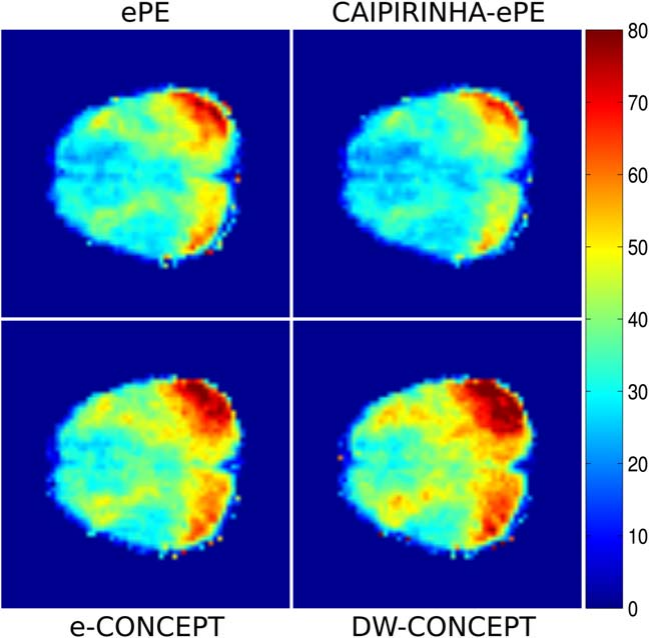
SNR maps of volunteer #1. All SNRs were normalized to the measurement time of the corresponding method, and rescaled to the measurement time of CONCEPT to allow easier comparison.

Figure 4 shows sample inositol maps for all five volunteers for all four compared methods. The CONCEPT results are visually hardly distinguishable, differences are most likely visible for volunteer #4 where ringing artifacts are visible. Across all five volunteers CAIPIRINHA‐ePE resulted in metabolic maps of much lower quality compared to the other three methods. One consequence of the SNR differences is visible in Figure 5, which shows the CRLB maps of inositol calculated by LCModel. Clearly DW‐CONCEPT has the smallest CRLB values of all accelerated methods. The wide range of mapped metabolites is demonstrated in Figure 6 which shows maps for total N‐acetyl‐aspartate, total creatine, total choline, glutamine + glutamate, and inositol of volunteer #2. In Figure 7 and Supporting Figure S1 spectra of volunteer #1 obtained at three different brain positions are shown for all methods. Although we observed baseline distortions in some spectra closer to the skull (see Supporting Fig. S1), high spectral resolution and improved fitting quality ensured a good mapping of all major metabolites. SNR efficiencies were compared in Table 2, demonstrating the efficiency of CONCEPT combined with DW. The resulting SNRs and relative SNRs for the proposed and the other methods are shown. DW‐CONCEPT performed best, resulting in a +9.5% higher SNR per unit time than e‐CONCEPT (*P* = 0.002), a +24.4% gain compared to ePE (*P* < 0.001) and a +39.7% gain compared to CAIPIRINHA‐ePE (*P* < 0.001). All measured SNR gains perfectly match the theoretically derived values in the Appendix section, which are +9.0% for DW‐ vs. e‐CONCEPT, and +25.2% for DW‐CONCEPT vs. ePE. As expected, ePE had about +12.4% higher SNR per unit time than CAIPIRINHA‐ePE (*P* = 0.002), likely due to g‐factor losses 15. SNR maps for a single volunteer are shown in Figure 8. Further improvement of the metabolic maps can be achieved by using L2 regularization, as illustrated for the total N‐acetyl‐aspartate map in Supporting Figure S2. Supporting Figure S3 shows lipid/total N‐acetyl‐aspartate ratio maps for the first volunteer for all methods.

**Table 2:** Mean and Standard Deviation for SNRs and for Relative SNRs (Averaged Over All 64 × 64 Voxels) of Each Volunteer for All Methods. DW‐CONCEPT Results in the Most SNR Efficient Readout Scheme. A Paired 2‐Tailed *t*‐test With Bonferroni Correction Yielded *P*‐Values of *P* < 0.05 Across All Methods. All SNRs were Normalized to the Measurement Time of the Corresponding Method, and Rescaled to the Measurement Time of CONCEPT to Allow Easier Comparison. The Measured SNR Gains Match the Theoretically Derived Values in the Appendix Section and are Therefore Displayed With Higher Precision.

Mean of SNRs	Vol 1	Vol 2	Vol 3	Vol 4	Vol 5	
ePE	36.23 ± 11.63	33.40 ± 8.21	38.32 ± 8.98	34.15 ± 11.30	27.66 ± 5.79	
CAIPIRINHA‐ePE	32.32 ± 9.91	29.91 ± 6.86	34.04 ± 8.41	30.71 ± 10.56	25.13 ± 6.18	
e‐CONCEPT	41.29 ± 12.86	37.45 ± 9.50	43.33 ± 9.59	37.50 ± 12.03	31.29 ± 6.33	
DW‐CONCEPT	44.95 ± 14.48	40.43 ± 10.18	46.64 ± 10.32	41.78 ± 13.76	33.86 ± 7.19	
Mean of rel. SNRs						Mean over Vol
$\frac{e‐\text{CONCEPT}}{\text{ePE}}$	1.17 ± 0.27	1.13 ± 0.17	1.15 ± 0.21	1.12 ± 0.25	1.16 ± 0.24	1.145 ± 0.017
$\frac{\text{DW}‐\text{CONCEPT}}{\text{ePE}}$	1.26 ± 0.31	1.22 ± 0.18	1.24 ± 0.22	1.24 ± 0.24	1.25 ± 0.27	1.244 ± 0.014
$\frac{e‐\text{CONCEPT}}{\text{CAIPIRINHA}‐\text{ePE}}$	1.29 ± 0.25	1.26 ± 0.22	1.30 ± 0.25	1.26 ± 0.29	1.30 ± 0.39	1.284 ± 0.021
$\frac{\text{DW}‐\text{CONCEPT}}{\text{CAIPIRINHA}‐\text{ePE}}$	1.41 ± 0.37	1.36 ± 0.23	1.40 ± 0.28	1.40 ± 0.31	1.41 ± 0.43	1.397 ± 0.017
$\frac{\text{DW}‐\text{CONCEPT}}{e‐\text{CONCEPT}}$	1.09 ± 0.23	1.08 ± 0.10	1.08 ± 0.12	1.12 ± 0.18	1.09 ± 0.22	1.094 ± 0.015
$\frac{\text{ePE}}{\text{CAIPIRINHA}‐\text{ePE}}$	1.12 ± 0.14	1.11 ± 0.10	1.13 ± 0.12	1.13 ± 0.14	1.13 ± 0.22	1.124 ± 0.007

## DISCUSSION

The proposed DW‐CONCEPT sampling approach provides SNR‐efficient acceleration with an optimized PSF to reduce signal leakage for full‐slice high‐resolution (i.e., 64 × 64) metabolic mapping at 7T in clinically attractive scan times of 5–6 min.

Recent reports on high‐resolution single‐slice (up to 128 × 128 matrix) and multi‐slice MRSI (up to 100 × 100) at 7T 11 and 9.4T 12 provide evidence of an increased need for SNR‐efficient accelerated sampling. This has led to a reduction of TR, if permitted by SAR constraints 11, 43, and the development of advanced parallel imaging methods (yet restricted to accelerations of ≤10) 15. While TR‐reduction and parallel imaging can somehow alleviate scan time problems, potentially even faster SSE approaches have not been established at $B_{0}\geq 7$ T due to high gradient hardware requirements 10, but are still under development. In particular the development of DW‐SSE approaches is highly desirable when Hamming filtering is targeted to make the use of SNR‐inefficient post‐acquisition k‐space Hamming filtering obsolete.

### Comparison of Acceleration Approaches: Sampling Density

Post‐acquisition Hamming filtering has been extensively used to minimize Gibbs ringing artifacts for ePE‐MRSI, but it is SNR inefficient. Optimal SNR efficiency can be achieved via acquisition weighting, but only if scan time restrictions permit the sampling of several averages 17. This is not the case for recent high‐resolution 7T and 9.4T ePE‐MRSI, which is time‐consuming already with short TR and a single average. At $B_{0}<7$ T some SSE approaches are even better suited for DW k‐space acquisition than acquisition weighting. For instance, spiral trajectories can be tailored to shape a Hanning filter without the need for averaging 27. Yet, this prolongs the spiral trajectory, and therefore requires more temporal or angular interleaves to maintain the same spectral bandwidth. Temporal interleaves cause spectral artifacts, and usually cannot be increased further at 7T. Increasing the angular interleaves further for high‐resolution spiral MRSI at 7T is also problematic, since this would transform the spiral trajectory into an almost radial k‐space readout. Therefore, spirals are very well suited for matching low‐pass k‐space filters at $B_{0}=3$ T, but are not well suited when using very high spatial resolutions at 7T. EPSI can be adapted to achieve a Hamming filter in the phase‐encoded direction by changing the phase encoding step size, or by acquisition weighting, at the expense of an increased scan time. In the frequency encoding direction, however, the same problems occur as for high‐resolution spirals at 7T. Various k‐space densities can also be accomplished by tweaking acquisition parameters of rosette trajectories, but achieving a Hamming filter is impossible due to inherent high‐pass filter contributions 20, 21. In contrast, the natural density of e‐CONCEPT is already a low‐pass filter, and filters like a Hamming or Hanning filter are therefore efficiently accomplished. As shown here, this can also be achieved very conveniently by adapting the radii of the circles. Thereby, any rotational invariant target filter can be achieved without altering the trajectory or gradient demands and with less additional scan time necessary. The optimization of the PSF via DW always leads to increased scan times. This is also the case in weighted averaging, as proposed by Pohmann and Von Kienlin 17. In both DW approaches, weighted averaging and our approach, the scan times and the resulting SNRs should be very similar. To subsume, our results show that DW‐CONCEPT achieves full‐slice high‐resolution 64 × 64 FID‐MRSI at 7T, both, in clinically attractive 5–6 min and with optimized SNR.

### Comparison of Acceleration Approaches: SNR Efficiency

Previous MRSI studies have often sacrificed SNR efficiency for speed. For instance, recent FID‐MRSI studies at $B_{0}\geq 7$ T have reduced SNR efficiency due to both increasing *g*‐factors associated with high parallel imaging acceleration 15 and the application of post‐acquisition Hamming filtering 25, 26. Our results show that DW‐CONCEPT leads to an +39.7% increased SNR efficiency even when compared to a fairly modest 5‐fold acceleration using efficient CAIPIRINHA parallel imaging and retrospective Hamming filtering. This is in agreement with previous studies 17. If permitted by SAR constraints, TR‐reduction is the most SNR‐efficient way of acceleration, since shorter TRs at Ernst angles even lead to a small increase in SNR efficiency, e.g., by about 4% when reducing the TR from 2 to 0.6 s with a *T*_1_ of 1.86 s 17. However, this is at the expense of increasingly strong *T*_1_‐weighting, which makes accurate metabolite quantification challenging 17. Spiral and EPSI have reduced SNR efficiencies for high resolution MRSI at 7T due to the rewinding gradients. If ramp‐sampling is performed, this problem is alleviated, but the k‐space density is strongly different from a low‐pass filter, which again decreases SNR efficiency for a Hamming target filter. Rosette trajectories have decreased SNR efficiencies due to k‐space densities that cannot be adapted to conventional k‐space filters. In contrast, e‐ and DW‐CONCEPT have no gradient rewinders, and at the same time can be easily adapted to rotational invariant filters. Further, we showed that DW‐CONCEPT reaches even a +24.4% higher SNR efficiency than the very slow gold standard ePE‐MRSI. This is consistent with recent reports by Furuyama et al., who concluded that already CONCEPT without DW and with a constant target k‐space density performed about equally well as EPSI in terms of SNR at 3T 22. The higher SNR obtained by the modified Pipe‐Menon density compensation compared to the usual method where the acquired (but already weighted) k‐space is flattened before gridding and Hamming filtering, could be explained by two possible effects: (a) The modified Pipe‐Menon density compensation does not result in a deviation of the targeted density as seen in Figure 1 and therefore the post‐gridding density compensation performs better and/or (b) Pre‐gridding density compensation of an already weighted k‐space to a flat k‐space together with post‐gridding filtering is in general an unnecessary step since merely compensating for the missing weights might be a better option SNR‐wise.

### Comparison of Acceleration Approaches: Maximum Speed

Most acceleration approaches differ in their maximum possible acceleration. Speed‐up factors are additionally influenced by scan parameters including in particular the spectral bandwidth and spatial resolution. Spiral MRSI is one of the fastest trajectories, since it applies acceleration in two spatial dimensions simultaneously. If a low spectral bandwidth of 1.8 kHz can be used, e.g., if lipid signals are taken care of in another way, high accelerations of 60–70 can be achieved even at 7T for high‐resolution MRSI using matrix sizes of 64 × 64 to 100 × 100. Very similar accelerations of 45–75 are possible via e‐CONCEPT. The maximum possible speed‐up for EPSI is exactly two times lower than for e‐CONCEPT 22. While at 7T with state‐of‐the‐art gradients, matrix sizes up to 64 × 64 can still be achieved with EPSI, a 100 × 100 matrix can only be realized with unacceptable SNR‐penalties 44. However, first approaches to overcome this limitation via k‐space readout segmentation have recently emerged 45, but reduce the acceleration even further. Rosettes can achieve the highest spatial resolutions within fixed gradient hardware limits compared to EPSI, spirals and CONCEPT, but the maximum acceleration is 2–4 times lower than for spirals 21. While for rosettes some of the excessive acceleration can be traded for decreased stress on the gradient hardware, DW‐CONCEPT can trade speed against further optimized SNR‐efficiency. Our results show that an optimal PSF can be reached via an 8‐fold increase of scan time from 40 s to 5 min 25 s, but this trade‐off can be balanced as desired. Similarly, PSF improvements via variable density spirals lead to increased scan times by the need for more angular or temporal interleaves 27.

### Comparison of Acceleration Approaches: Artifact Susceptibility

The different trajectories also vary in their susceptibility to artifacts. EPSI at $B_{0}=3$ T is fairly robust to artifacts, which has certainly contributed to its dissemination 16, 46, 47, but with increasing gradient stress at higher *B*_0_s, strong frequency drifts and gradient inaccuracies may result in new challenges. Spiral trajectories are inherently less sensitive to motion 48 and less gradient‐demanding, but more prone to system imperfections 23. CONCEPT is less sensitive to hardware imperfections that cause frequency drifts, eddy currents and inaccurate trajectories 23. DW‐CONCEPT additionally improves Gibbs ringing without the need for strong spatial filters. In contrast parallel imaging is immune to gradient imperfections due to low gradient requirements. On the other hand, parallel imaging can introduce additional lipid artifacts that are absent with SSE. Overall, our phantom and in vivo results show no major artifacts for DW‐CONCEPT, except for some baseline variability, which was efficiently handled by LCModel spline baseline correction and should be further investigated.

### Limitations

Possible deviation of SSE trajectories due to system imperfections can result in spatial and spectral artifacts. Previous reports have shown that CONCEPT is more robust than other approaches 23. Nevertheless, a better characterization and correction may lead to further improvements. In principle our gradient hardware would allow matrix sizes up to 100 × 100 with a spectral bandwidth of 2778 Hz, but due to acoustic noise our 64 × 64 matrix was chosen fairly conservative. Further improvement in spatial resolution also for other SSE approaches would benefit from a reduction of gradient noise or rearrangement of the circles. It is important to note that our gradient system has forbidden acoustic frequencies at 550 ± 50 and 1100 ± 150 Hz, which limits the choice of the spectral bandwidth with the risk of severe gradient damage when ignored. At ≤ 9.4 T high‐resolution MRSI via CONCEPT may be challenging due to further increased gradient demands. For even higher *B*_0_s and spatial resolutions, rosette trajectories may be a better alternative 21. SSE can cause substantial frequency drifts over time, which was not corrected for in this study. However, promising approaches such as the use of interleaved navigators have been proposed and will be implemented in future sequence versions 49, 50. Concerning the metabolic maps we observe a stronger gray/white matter contrast for the CONCEPT sequences which does not appear for the Cartesian sequences and also not on any ratio map. This will be investigated in the future, together with a possibility of reducing the measurement time for DW‐CONCEPT by trading *N*_min_ against SNR‐efficiency.

## CONCLUSION

In conclusion, we have developed and evaluated an SSE approach for MRSI at 7T that combines SNR‐efficiency and PSF‐optimization with time‐efficient sampling. Thereby, we achieved full‐slice high‐resolution metabolic mapping of a large number of chemical compounds via FID‐MRSI in clinically attractive scan times of 5–6 min.

## Supporting information


**Fig. S1**. Comparison of spectra taken from three representative voxels of the first volunteer without lipid regularization. Spectra were taken from LCModel and show the fitting (red) and the measured data (black).
**Fig. S2**. A L2 lipid regularization shows that lipid suppression can further enhance the quality of the metabolic maps as visualized in the tNAA map for the third volunteer. Spectra before (blue) and after (red) regularization of a chosen voxel (green) are shown on the right.
**Fig. S3**. Lipid/tNAA ratio maps of volunteer #1 scaled in arbitrary units. The tNAA maps were taken from the LCModel results, while the lipids were quantified by integration between 0.7 and 1.7 ppm. The baseline variations could be corrected by subtraction of the spectra from polynomial fits (up to the 6th order, not including the water peak). The reconstruction of the data was according to the in vivo protocol, e.g. all maps created resulted from a Hamming weighted k‐space. The ringing artifacts occurring in the ePE and CAIPIRINHA‐ePE maps likely result from movement, while additional artifacts for CAIPIRINHA‐ePE may be explained by aliasing, in contrast to e‐ and DW‐CONCEPT.

## APPENDIX A

This appendix describes how the theoretical SNR increases of 114.9% and 125.2% for the e‐CONCEPT and DW‐CONCEPT k‐space density relative to a uniform density can be calculated. When combining Eqs. (1), (2), we get:

(A1) $$\frac{{\text{SNR}}_{\text{Acq}}}{{\text{SNR}}_{\text{Uniform}}}=\sqrt{\frac{|\sigma_{\text{Uniform}}{|}^{2}}{|\sigma_{\text{Acq}}{|}^{2}}}=\sqrt{\frac{\int d^{n}k\frac{\rho_{\text{Target}}^{2}}{\rho_{\text{Uniform}}}}{\int d^{n}k\frac{\rho_{\text{Target}}^{2}}{\rho_{\text{Acq}}}}}$$

To solve this, we need to define the three different densities e‐CONCEPT, uniform, and Hamming:

(A2) $$\rho_{e‐\text{CONCEPT}}=\frac{d_{1}}{k}$$

(A3) $$\rho_{\text{Uniform}}=d_{2}$$

(A4) $$\rho_{\text{Target}}=\rho_{\text{DW}‐\text{CONCEPT}}=d_{3}\cdot (\alpha +\beta \cos(\pi k/k_{\text{max}})).$$

To assure a fair comparison, we require all k‐space densities to be measured during the same measurement time *T*_Meas_:

(A5) $$T_{\text{Meas}}=\int d^{2}k\rho_{e‐\text{CONCEPT}}=\int d^{2}k\rho_{\text{Uniform}}=\int d^{2}k\rho_{\text{DW}‐\text{CONCEPT}}.$$

Using this equation, we can calculate the constants *d*_1_, *d*_2_, and *d*_3_. For e‐CONCEPT:

(A6) $$T_{\text{Meas}}=\int d^{2}k\rho_{e‐\text{CONCEPT}}=\int_{0}^{2\pi }\int_{0}^{k_{\text{max}}}dkdk_{\phi }\;k\frac{d_{1}}{k}=2\pi k_{\text{max}}d_{1}.$$

For the uniform k‐space:

(A7) $$T_{\text{Meas}}=\int d^{2}k\rho_{\text{Uniform}}=\int_{0}^{2\pi }\int_{0}^{k_{\text{max}}}dkdk_{\phi }\;kd_{1}=\pi k_{\text{max}}^{2}d_{2}.$$

For the DW‐CONCEPT k‐space density:

(A8) $$\begin{array}{ll} & T_{\text{Meas}}=\int d^{2}k\rho_{\text{DW}‐\text{CONCEPT}}=\\ & \int_{0}^{2\pi }\int_{0}^{k_{\text{max}}}dkdk_{\phi }\;kd_{3}\cdot (\alpha +\beta \cos(\pi k/k_{\text{max}}))=\pi k_{\text{max}}^{2}(\alpha -\frac{4\beta }{\pi^{2}})d_{3}. \end{array}$$

Now with all constants calculated, we can solve Eq. (A1) for e‐CONCEPT:

(A9) $$\frac{{\text{SNR}}_{e‐\text{CONCEPT}}}{{\text{SNR}}_{\text{Uniform}}}=\pi \sqrt{\frac{3A_{1}}{2A_{2}}}\approx 1.149$$

and for DW‐CONCEPT:

(A10) $$\frac{{\text{SNR}}_{\text{DW}‐\text{CONCEPT}}}{{\text{SNR}}_{\text{Uniform}}}={\left(\alpha -\frac{4\beta }{\pi^{2}}\right)}^{-1}\sqrt{\frac{A_{1}}{2}}\approx 1.252$$

where $A_{1}:=(2\alpha^{2}+\beta^{2}-\frac{16\alpha \beta }{\pi^{2}})$ and $A_{2}:=(4\alpha^{2}\pi^{2}+\beta^{2}(3+2\pi^{2})-48\alpha \beta )$. Further

(A11) $$\frac{{\text{SNR}}_{\text{DW}‐\text{CONCEPT}}}{{\text{SNR}}_{e‐\text{CONCEPT}}}\approx 1.090$$

## APPENDIX B

This appendix describes how the solution of Eq. (6) can be found. We can rewrite and integrate Eq. (6) to

(B1) $$\int dKK\cdot (\alpha +\beta \cos(\pi K/k_{\text{max}}))=\int dkc_{1}$$

which yields

(B2) $$c_{1}k(K)=\alpha \frac{K^{2}}{2}+\beta \frac{{\left(k_{\text{max}}\right)}^{2}}{\pi^{2}}\cos(\pi K/k_{\text{max}})+\beta \frac{k_{\text{max}}K}{\pi }\sin(\pi K/k_{\text{max}})+c_{2},$$

where *c*_1_ is a proportionality constant and *c*_2_ the constant of integration. From Eqs. (5) and (B2) we obtain

(B3) $$\begin{array}{l} c_{1}=k_{\text{max}}\left(\frac{\alpha }{2}-\frac{2\beta }{\pi^{2}}\right),\\ \;c_{2}=-\frac{\beta {\left(k_{\text{max}}\right)}^{2}}{\pi^{2}}, \end{array}$$

which allows us to calculate *K*(*k*) by inverse series expansion. The most efficient way to sample a Hamming‐weighted k‐space uses the fewest number of circles *N*. This can be achieved by defining the first circle radius as $K(k_{1})=1/($2FOV) since then the weigths around the origin are minimized, which results in the lowest *N*_min_ necessary for Hamming weighting, see Table 1. With this definition the coordinates for density weighting read now

(B4) $$k_{i+1}=k\left(\frac{1}{2\text{FOV}}\right)+\frac{i}{N-1}\left(k_{\text{max}}-k\left(\frac{1}{2\text{FOV}}\right)\right),$$

for i=0,…,N−1, where k(K=1/(2FOV)) is given by Eq. (B2). The corresponding radii are then given by $K(k_{i+1})$. For e‐CONCEPT and DW‐CONCEPT the last circles were defined such that the same maximum k‐space extent is measured.

### Comments on *K*(*k*)

The analytical solution *K*(*k*) of Eq. (6) provides an easy to implement and computational fast approach for density weighting. Further it could be useful for the implementation of DW‐spirals, since the transformation to spiral coordinates (t,β) 30

(B5) $$\left(k_{x},k_{y})\to (K(t)\cos(g(t)\omega +\beta ),K(t)\sin(g(t)\omega +\beta )\right)$$

has the same Jacobian (which is independent of *g*(*t*)) and results therefore in the same solution. The function *K*(*t*) describes the radial increase of the spiral turnings, which become Archimedean for K(t)=g(t). The variables *ω* and *β* represent the angular velocity and the angular interleaves, respectively. For the sake of simplicity, gradient and slewrate constraints were not taken into account here.
