# Supplementary figures and images for: Density‐weighted concentric circle trajectories for high resolution brain magnetic resonance spectroscopic imaging at 7T
Source: Magn Reson Med. 2017 Nov 6;79(6):2874–85. doi: 10.1002/mrm.26987 (PMC5873433; doi:10.1002/mrm.26987)

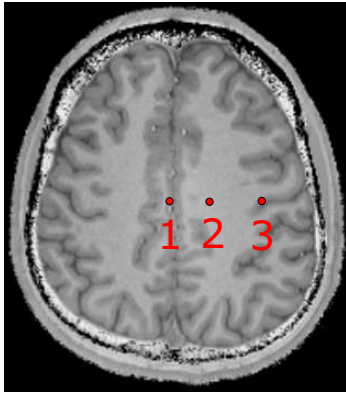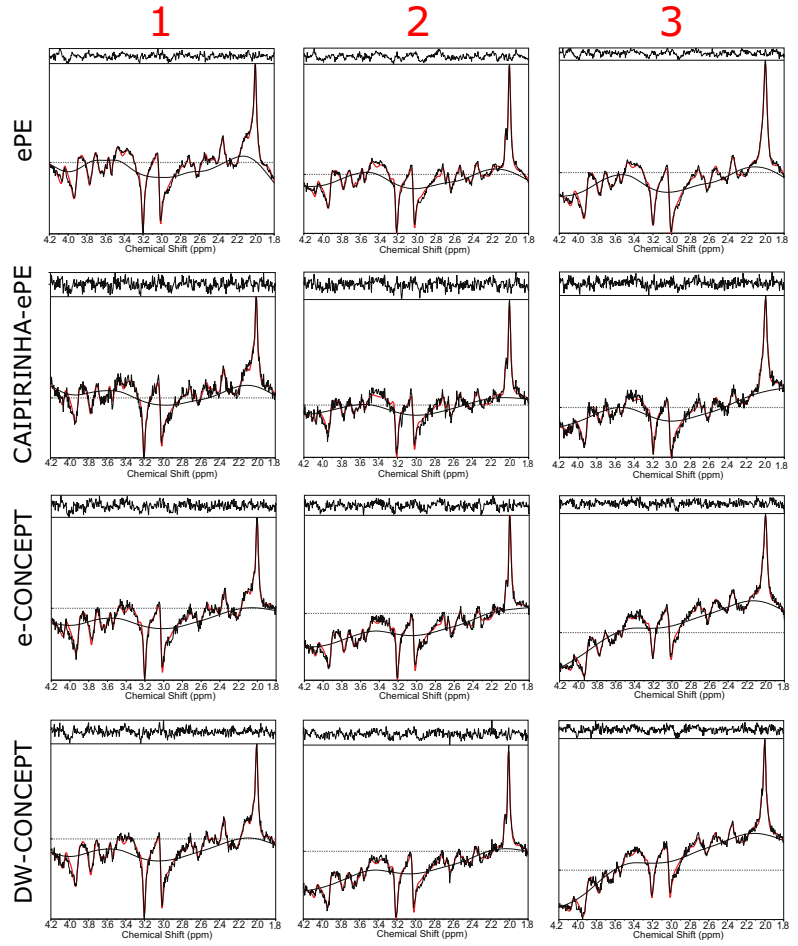

Supporting Figure S1

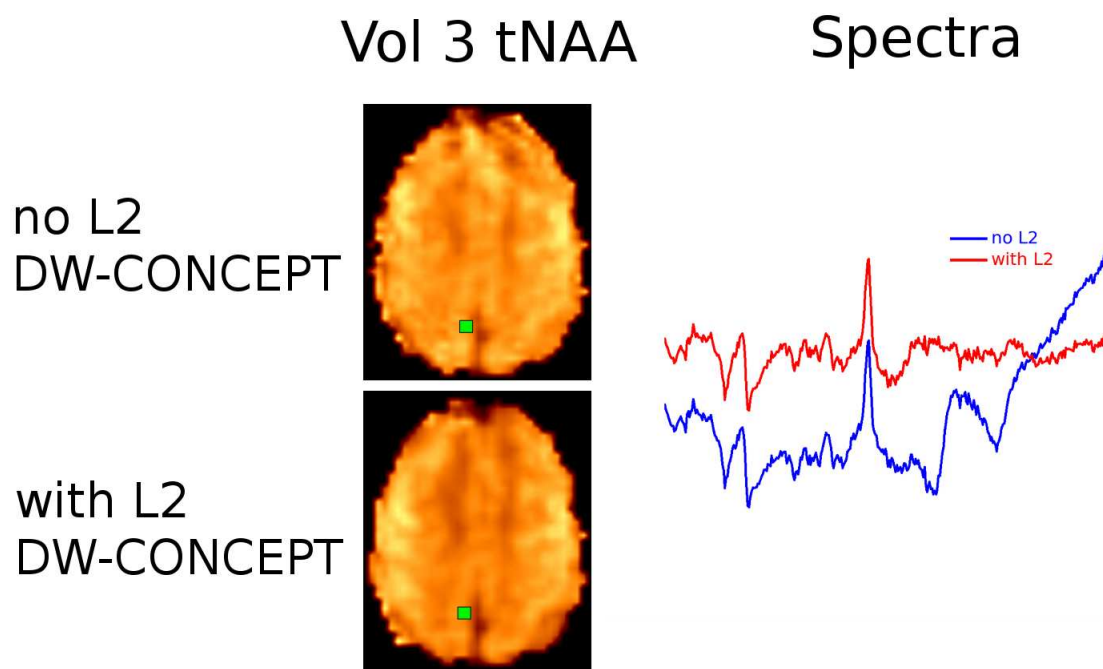

Supporting Figure S2

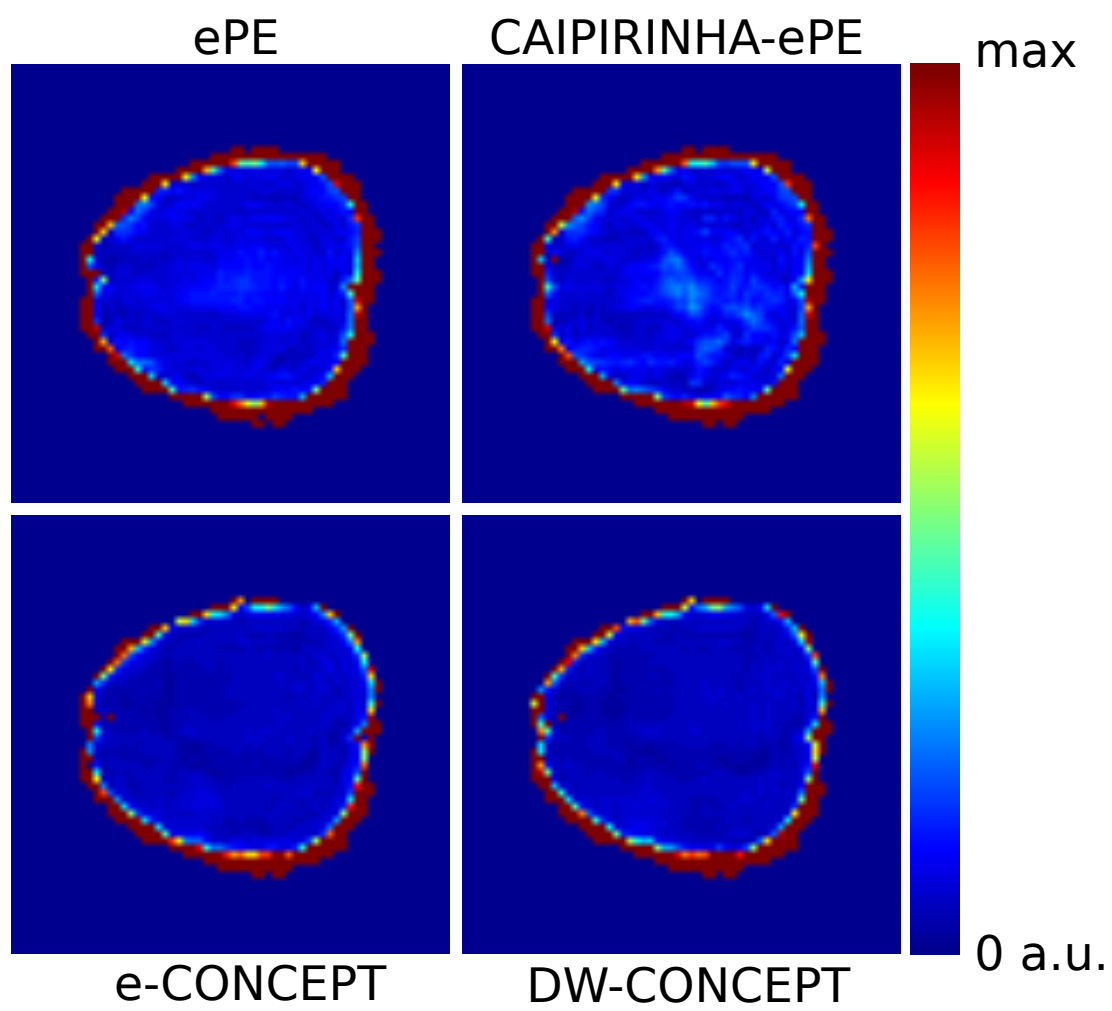

Supporting Figure S3

Supplement: Supplementary file 1 — Fig. S1. Comparison of spectra taken from three representative voxels of the first volunteer without lipid regularization. Spectra were taken from LCModel and show the fitting (red) and the measured data (black). Fig. S2. A L2 lipid regularization shows that lipid suppression can further enhance the quality of the metabolic maps as visualized in the tNAA map for the third volunteer. Spectra before (blue) and after (red) regularization of a chosen voxel (green) are shown on the right. Fig. S3. Lipid/tNAA ratio maps of volunteer #1 scaled in arbitrary units. The tNAA maps were taken from the LCModel results, while the lipids were quantified by integration between 0.7 and 1.7 ppm. The baseline variations could be corrected by subtraction of the spectra from polynomial fits (up to the 6th order, not including the water peak). The reconstruction of the data was according to the in vivo protocol, e.g. all maps created resulted from a Hamming weighted k‐space. The ringing artifacts occurring in the ePE and CAIPIRINHA‐ePE maps likely result from movement, while additional artifacts for CAIPIRINHA‐ePE may be explained by aliasing, in contrast to e‐ and DW‐CONCEPT. [file MRM-79-2874-s001.pdf]
